# S-1-based concurrent chemoradiotherapy in the treatment of locally advanced non-small cell lung cancer: A systematic review and meta-analysis protocol

**DOI:** 10.1097/MD.0000000000010397

**Published:** 2018-04-13

**Authors:** Feiyu Liu, Chaoyang Wang, Tao Hu, Wei Wang

**Affiliations:** a Department of Pharmacy; b Department of Thoracic Surgery, The Affiliated Yantai Yuhuangding Hospital of Qingdao University, Yantai, Shandong, China; c Department of Thoracic and Cardiovascular Surgery, The University of Texas MD Anderson Cancer Center, Houston, TX.

**Keywords:** chemoradiotherapy, CRT, non-small cell lung cancer, NSCLC, S-1

## Abstract

**Background:**

Lung cancer is the leading cause of cancer-related deaths in the world, and non-small cell lung cancer accounts for > 75% of all lung cancer cases. Cisplatin-based concurrent chemoradiotherapy has become the standard treatment for locally advanced non-small cell lung cancer (NSCLC). Third-generation chemotherapy agents plus cisplatin have been most commonly used in concurrent chemoradiotherapy, which is also associated with more adverse effects and acute toxicities. S-1 as an oral chemotherapeutic agent exhibits higher antitumor activity, less adverse effects, and better biological availability. Recently, studies illustrated S-1-based concurrent chemoradiotherapy also had excellent effects in the treatment of locally advanced NSCLC.

**Methods:**

A systematic literature search will be performed through February 2018 using MEDLINE, EMBASE, the Cochrane Central Register of Controlled Trials, and Google Scholar for relevant articles published in any language. Randomized controlled trials and prospective comparative studies will be included. All meta-analyses will be performed using Review Manager software. The quality of the studies will be evaluated using the guidelines listed in the Cochrane Handbook. The Preferred Reporting Items for Systematic Reviews and Meta-Analyses statements will be followed until the findings of the systematic review and meta-analysis are reported.

**Results:**

The results of this systematic review and meta-analysis will be published in a peer-reviewed journal.

**Conclusion:**

Our study will draw an objective conclusion of the efficacy and safety of S-1-based chemoradiotherapy in the treatment of locally advanced unresectable NSCLC and provides level I evidence for clinical decision makings.

## 1 Introduction

Lung cancer is the leading cause of cancer-related deaths in the world, and non-small cell lung cancer (NSCLC) accounts for > 75% of all lung cancer cases.^[1,2]^ Approximately 30% of all lung cancer patients are diagnosed with stage III disease,^[3]^ for whom chemotherapy and radiotherapy utilized alone or combinedly are mainly nonsurgical treatment options. Previous randomized phase III study has illustrated that concurrent chemoradiotherapy (CRT) has advantages over sequential chemoradiotherapy in terms of response and survival.^[4]^ Cisplatin-based CRT has become the standard treatment for locally advanced NSCLC.^[5–8]^ Nevertheless, third-generation chemotherapy agents plus cisplatin have been most commonly used in CRT, which is also associated with more adverse effects and acute toxicities compared with sequential chemoradiotherapy.^[9]^ Therefore, exploring new CRT regimens with better tolerance and lower toxicity for patients with NSCLC are desperately needed.

S-1 is a novel oral combination drug comprising tegafur (FT), a prodrug of 5-fluorouracil (5-Fu), and 2 modulators of 5-FU metabolism, gimeracil (CDHP), and oteracil (Oxo), in a 1:0.4:1 molar ratio (FT:CDHP:Oxo). Evidence suggested that S-1 exhibits higher antitumor activity, less adverse effects, and better biological availability while compared with conventional 5-FU,^[10–12]^ which can sensitize cancer cells to radiotherapy.^[13]^ In recent years, S-1 as an excellent CRT drug, single use or combined with platinum, has been widely applied for advanced NSCLC and achieved good clinical outcomes.^[14–20]^ However, the sample size of these studies was relatively small and results in weak statistical power. Therefore, we conduct a systematic review and meta-analysis related to S-1-based CRT versus non-S-1-based CRT in the treatment of advanced NSCLC to further evaluate the clinical value of S-1. Moreover, in order to minimize the heterogeneity and bias, we will select randomized controlled trials (RCTs) and prospective comparative studies. The evidence grade will be determined by using the guidelines of the Grading of Recommendations, Assessment, Development, and Evaluation (GRADE) system. If data are sufficient, we will also conduct subgroup analyses using different histological types.

## 2 Objective

A systematic review and meta-analysis will be performed to assess the efficacy and safety of S-1-based CRT in the treatment of patients with locally advanced NSCLC.

## 3 Methods

This protocol for systematic review and meta-analysis is performed according to the Preferred Reporting Items for Systematic Review and Meta-Analysis Protocols (PRISMA-P) statement.^[21]^ This protocol has been registered in the PROSPERO network (registration number: CRD42018087982). The systematic review and meta-analysis will be reported according to the Preferred Reporting Items for Systematic Reviews and Meta-Analyses (PRISMA) guidelines.^[22]^

## 4 Eligibility criteria

### 4.1 Types of participants

The included participants will be adults who were diagnosed with locally advanced NSCLC histologically or cytologically confirmed and treated with CRT. Comparisons of S-based CRT with non-S-1-based CRT in the clinical treatment were evaluated. There will be no restrictions regarding sex, race/ethnicity, education and economic status, and no restriction in publication language.

### 4.2 Types of studies

We propose to include studies that report comparisons between S-1-based CRT and non-S-1-based CRT in the treatment of locally advanced NSCLC. RCTs and prospective comparative studies will be used for the qualitative and quantitative synthesis of the systematic review.

### 4.3 Exclusion criteria

Non-peer-reviewed articles, review articles, case reports, case series, animal studies, meeting abstracts, letters to the editor, commentaries, editorials, proceedings, and other nonrelevant studies will be excluded from analysis.

### 4.4 Information sources

We will perform a systematic literature search through February 28, 2018 using MEDLINE, EMBASE, the Cochrane Central Register of Controlled Trials, and Google Scholar for relevant articles published in any language.

### 4.5 Search strategy

The relevant searching terms will match Medical Subject Heading terms, and the searches will be repeated immediately before the final analyses to identify additional studies for inclusion. An example of the PubMed search strategy is shown in Table 1.

**Table 1:**
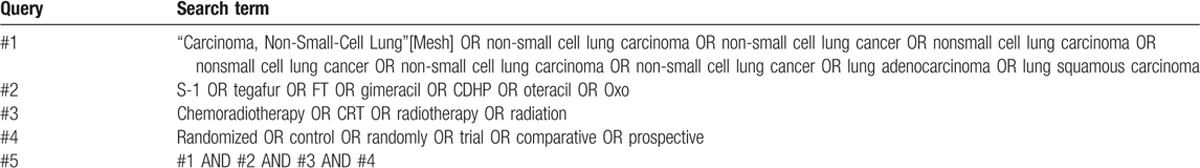
Search strategy for PubMed.

### 4.6 Study records

#### 4.6.1 Selection of studies

Two review authors (FL and CW) will independently screen titles and abstracts of all the potential studies to assess whether they meet the inclusion criteria as defined by the protocol. We will retrieve the full text of all potentially eligible studies and 2 review authors (FL and CW) will independently screen the full-text and identify studies for inclusion, and record reasons for exclusion of the ineligible studies. Any disagreement will be resolved through discussion or, if required, consultation with a third review author (TH or WW). Duplicates will be excluded and multiple reports of the same study will be integrated into one unit of interest in the review. The selection process will be recorded in sufficient detail to complete a PRISMA flow diagram and “Characteristics of excluded studies” table.^[23]^ No language restrictions will be imposed.

#### 4.6.2 Data extraction and management

Data will be extracted from the included studies by 3 authors (FL, CW, and TH) independently and recorded on a predesigned data collection form. We will extract the following study characteristics:

(1) *Study characteristics:* study design, number of study centers and locations, study setting, withdrawals, total duration of the trial, periods of data collection, follow-up duration, and blanking periods.
(2) *Population characteristics:* inclusion and exclusion criteria, number, mean age, age range, gender, diagnostic criteria, pathological confirmation, staging of the tumor according to the IASLC (International Association for the Study of Lung Cancer) TNM classification for lung cancer.
(3) *Intervention characteristics:* total radiation dose, fractions, chemotherapy agents dose, administration frequency, and cycles.
(4) *Outcomes:* primary and secondary outcomes specified and collected, and time points reported.

#### 4.6.3 Outcomes

##### 4.6.3.1 Primary outcome

The primary outcome measure of our systematic review is overall survival (OS).

##### 4.6.3.2 Secondary outcomes

The secondary outcomes are: objective response rate (ORR), progression-free-survival (PFS), grade 3 and 4 adverse events.

##### 4.6.3.3 Assessment of risk of bias

Three review authors (FL, CW, and TH) will independently assess the risk of bias for each study using the criteria outlined in the Cochrane Handbook for Systematic Reviews of Interventions. Any disagreements will be resolved by discussion or by involving another review author (WW). The risk of bias will be assessed according to the following domains: random sequence generation; allocation concealment; blinding of participants and personnel; blinding of outcome assessment; incomplete outcome data; selective outcome reporting; and other bias. Each potential source of bias will be graded as high, low, or unclear and a quote from the study report with a justification for our judgement will be provided in the “Risk of bias” table. The risk of bias judgements across different studies for each of the domains listed will be summarized.

##### 4.6.3.4 Data synthesis

Data from studies judged to be clinically homogeneous will be pooled using Review Manager 5.3 software. Heterogeneity between studies will be assessed using the Cochran's *Q* and Higgins *Ι*^2^ statistic. *P* < .10 for the Chi^2^ statistic or an *I*^2^ > 50% will be considered as showing considerable heterogeneity, and the data will be analyzed using the random-effect model. Otherwise, the fixed-effect model will be used. The Mantel–Haenszel method will be applied for pooling of dichotomous data and results will be presented as relative risk (RR) with their 95% confidence intervals (CI). Inverse variance method will be used for pooling of continuous data and results will be presented as standardized mean difference (SMD) with their 95% CI.

##### 4.6.3.5 Subgroup analysis

If data are sufficient, we will conduct subgroup analyses on different histological types: adenocarcinoma and squamous carcinoma. Subgroup analyses will also be performed to explore potential sources of heterogeneity.

##### 4.6.3.6 Sensitivity analysis

A sensitivity analysis will be performed to confirm whether the pooled results are robust and credible by excluding highly biased studies.

##### 4.6.3.7 Dealing with missing data

In the condition of missing or unclear data, study authors will be contacted at the eligibility assessment and/or data extraction stage. Secondary publications may be considered as missing data if they have the same study population.

##### 4.6.3.8 Publication bias

Egger's regression test will be performed to assess the publication bias of the included studies.^[24]^ If there is a publication bias, trim and fill analysis will be performed.

##### 4.6.3.9 Evidence evaluation

The evidence grade will be determined by using the guidelines of the Grading of Recommendations, Assessment, Development, and Evaluation (GRADE) system, and using 4 levels—high quality, moderate quality, low quality, and very low quality.^[25]^

## 5 Discussion

S-1 is a promising chemotherapy agent with good efficacy and acceptable tolerability in various solid tumors, such as advanced gastric cancer,^[26]^ colorectal cancer,^[10]^ esophageal cancer,^[27]^ NSCLC,^[12]^ pancreatic cancer,^[11]^ and head and neck cancer.^[28]^ And as mentioned above, S-1 has also exhibited excellent effects as a CRT regimen for locally advanced NSCLC. This protocol presents the methodology of a systematic review for assessing the efficacy and safety of S-1 combined with radiotherapy in the treatment of advanced unresectable NSCLC. We will comprehensively search, screen, assess, and extract valuable data from several databases as previously mentioned, and report this review results according to the PRISMA guidelines. To our knowledge, this will be the first systematic review and meta-analysis comparing the efficacy and safety of S-1-based CRT with non-S-1-based CRT in the treatment of locally advanced NSCLC.

## Author contributions

FL, CW, and WW conceived and designed this study. FL and CW drafted the protocol. FL, CW and TH will conduct the search, data screening and extraction. FL, CW, TH and WW have critically reviewed the manuscript and approved it for publication.

**Conceptualization:** Feiyu Liu, Chaoyang Wang.

**Data curation:** Feiyu Liu, Chaoyang Wang.

**Formal analysis:** Feiyu Liu, Chaoyang Wang.

**Funding acquisition:** Wei Wang.

**Investigation:** Feiyu Liu.

**Methodology:** Feiyu Liu, Chaoyang Wang.

**Project administration:** Chaoyang Wang.

**Resources:** Wei Wang.

**Software:** Feiyu Liu, Chaoyang Wang.

**Supervision:** Wei Wang, Tao Hu.

**Validation:** Tao Hu.

**Visualization:** Tao Hu.

**Writing – original draft:** Wei Wang, Feiyu Liu, Chaoyang Wang.

**Writing – review & editing:** Wei Wang, Feiyu Liu, Chaoyang Wang.
